# Antiretroviral Simplification with Darunavir/Ritonavir Monotherapy in Routine Clinical Practice: Safety, Effectiveness, and Impact on Lipid Profile

**DOI:** 10.1371/journal.pone.0037442

**Published:** 2012-05-29

**Authors:** José R. Santos, José Moltó, Josep M. Llibre, Eugenia Negredo, Isabel Bravo, Arelly Ornelas, Bonaventura Clotet, Roger Paredes

**Affiliations:** 1 Lluita contra la SIDA Foundation, Hospital Universitari Germans Trias i Pujol, Badalona, Barcelona, Spain; 2 Universitat Autònoma de Barcelona, Barcelona, Spain; 3 Department of Econometrics, Statistics and Spanish Economy, University of Barcelona, Barcelona, Spain; 4 IrsiCaixa Foundation, Barcelona, Spain; University of Pittsburgh, United States of America

## Abstract

**Background:**

Simplification of antiretroviral treatment (ART) with darunavir/ritonavir (DRV/r) monotherapy has achieved sustained suppression of plasma viral load (pVL) in clinical trials; however, its effectiveness and safety profile has not been evaluated in routine clinical practice.

**Methodology/Principal Findings:**

We performed a retrospective cohort analysis of HIV-1-infected patients who initiated DRV/r monotherapy once daily with a pVL <50 copies/mL under ART and at least 1 subsequent follow-up visit in our clinic. The primary study endpoints were the percentage of patients with virological failure (VF, defined as 2 consecutive pVL>50 copies/mL) at week 48, and time to VF. Other causes of treatment discontinuation and changes in lipid profile were evaluated up to week 48. Ninety-two patients were followed for a median (IQR) of 73 (57–92) weeks. The median baseline and nadir CD4+ T-cell counts were 604 (433–837) and 238 (150–376) cells/mm3, respectively. Patients had previously received a median of 5 (3–9) ART lines and maintained a pVL<50 copies/mL for a median of 76 (32–176) weeks before initiating DRV/r monotherapy. Nine (9.8%) patients developed VF at week 48; time to VF was 47.1 (IQR: 36.1–47.8) weeks among patients with VF. Other reasons for changing ART were gastrointestinal disturbances (n = 3), rash (n = 1), and impaired CD4 recovery (n = 2). Median low-density lipoprotein cholesterol levels increased from 116.1 mg/dL at baseline to 137.3 mg/dL at 48 weeks (p = 0.001).

**Conclusions/Significance:**

Treatment simplification with DRV/r monotherapy seems safe and effective in routine clinical practice. Further research is needed to elucidate the effect of DRV/r monotherapy on cholesterol levels.

## Introduction

Standard-of-care antiretroviral therapy (ART) combines at least 3 antiretroviral drugs including 2 nucleos(t)ide reverse transcriptase inhibitors (NRTIs) [1], which may be associated with toxicity arising from mitochondrial dysfunction [2]. NRTI-sparing strategies could potentially be as effective as standard ART, while proving less toxic and preserving future treatment options.

Monotherapy with protease inhibitors (PIs) is particularly attractive as an NRTI-sparing strategy, especially in cases of NRTI-related toxicity or intolerance, and its use in these settings is still recognized in some guidelines [3], [4]. Darunavir/ritonavir (DRV/r) may be particularly suited for PI monotherapy, because it has a high genetic barrier, a favorable safety and pharmacokinetic profile, and can be administered once daily [5], [6]. Randomized clinical trials have shown that DRV/r monotherapy as a simplification strategy has similar efficacy to triple ART, with reduced costs and low rates of resistance [7]–[9]. However, there are concerns that PI monotherapy might be associated with viral evolution in the central nervous system or other compartments with different degrees of penetration, and neurocognitive impairment [10]. Duration of the response to PI monotherapy is unclear, as are the clinical factors associated with virological failure, particularly in routine clinical practice, where the efficacy and safety profile of DRV/r remains largely unknown.

The objective of this study was to evaluate the effectiveness and safety profile of darunavir/ritonavir (DRV/r) monotherapy as an NRTI-sparing treatment simplification strategy in HIV-infected patients with sustained viral suppression in routine clinical practice.

## Methods

Our study sample comprised all consecutive patients who had initiated DRV/r monotherapy with an HIV-1 RNA load <50 copies/mL between December 2007 and January 2010. Data were retrieved from a prospectively compiled database (electronic medical records). All selected patients were followed until the last one had completed 48 weeks of follow-up or switched DRV/r monotherapy. Patients started with DRV/r 900/100 mg once daily (Prezista®, Tibotec a division of Janssen-Cilag International, Beerse, Belgium) and were subsequently switched to DRV/r 800/100 mg once daily when 400-mg tablets became commercially available. Patients who had voluntarily discontinued their therapy or who had been lost to follow-up before completing 48 weeks were not considered eligible for the analysis, but were included for the full dataset effectiveness analysis. Demographic and clinical characteristics, viral load, CD4+ T-cell count, creatinine, liver enzymes, and fasting lipid profile (total cholesterol, high-density lipoprotein [HDL] cholesterol, low-density lipoprotein [LDL] cholesterol, and triglycerides) were recorded when monotherapy was started (baseline) and every 12 weeks thereafter. In addition, adverse events leading to discontinuation of treatment and the development of resistance mutations in those patients whose DRV/r monotherapy failed were also evaluated. The study was approved by the ethics committee from Hospital Germans Trias i Pujol, Badalona, Spain, and it was performed according to the stipulations of the Declaration of Helsinki (Seoul, 2008). All patients gave their written informed consent for their medical information to be used in scientific research.

The primary endpoints of the study were the proportion of patients whose DRV/r monotherapy had failed at 48 weeks and their time to virological failure. Virological failure was defined as an increase in viral load >50 copies/mL at 2 consecutive determinations. The first date with HIV-1 viral load >50 copies/mL was used to calculate the time to virological failure. The secondary endpoints were the percentage of patients who discontinued their treatment for any reason, time to treatment discontinuation, the overall percentage of patients who maintained viral suppression, and development of protease resistance mutations. Changes in monotherapy because of toxicity, virological failure, or other patient- or physician-based reasons were recorded to calculate the time and treatment discontinuation rate. Factors associated with virological failure, the percentage of patients with blips, time to blips, and changes in CD4+ T-cell count, creatinine level, and liver and fasting lipid profiles were also analyzed.

To assess the impact of DRV/r monotherapy on lipid profile, we analyzed overall changes in fasting lipid profile from baseline. We also performed sub-analyses according to previous use of PIs, tenofovir (TDF), abacavir (ABC), and other NRTIs, and compared the proportion of patients with dyslipidemia, risk factors associated with dyslipidemia and lipid-lowering drug use at baseline and 48 weeks. Dyslipidemia was defined according to the criteria of the National Cholesterol Education Program (NCEP) Expert Panel on Detection, Evaluation, and Treatment of High Blood Cholesterol in Adults (Adult Treatment Panel III) parameters [11], as well as to the use of lipid-lowering drugs (ezetimibe, statins, and fibrates).

**Table 1:** Baseline characteristics.^a^

	General cohort (n = 92)
Male	62 (67.4)
Age (years)^b^	44.4 (38.8–49.9)
HCV co-infection	22 (23.9)
CDC A	76 (82.6)
Time since HIV diagnosis (years)^b^	13.2 (8.3–18.1)
Time on treatment (years)^b^	10.4 (5.2–14.1)
Type of toxicity^a^	
Dyslipidemia	20 (21.7)
Kidney	6 (6.5)
Gastrointestinal	5 (5.4)
Central nervous system	2 (2.2)
Liver	1 (1.1)
Peripheral neuropathy	1 (1.1)
Jaundice	1 (1.1)
Others	5 (5.4)
No. of prior ARV regimens^b^	5 (3–9)
No. of prior PIs^b^	2 (1–3)
ARV drugs use at entry	
3TC/FTC	70 (76.1)
TDF	52 (56.5)
ABC	18 (19.6)
Other NRTIs	7 (7.6)
LPV	32 (34.8)
ATV	27 (29.3)
FosAPV	10 (10.9)
DRV	9 (9.8)
SQV	5 (5.4)
NNRTIs	9 (9.8)
RAL	2 (2.2)
CD4+ nadir (cells/mm^3^)^b^	238 (150–376)
CD4+ T cell count (cells/mm^3^)^b^	604 (433–837)

a All values are expressed as No. (%), unless otherwise indicated.

b Median (interquartile range).

Abbreviations: HCV, hepatitis C virus; CDC, Centers for Disease Control and Prevention; ARV, antiretroviral; 3TC/FTC, lamivudine/emtricitabine; TDF, tenofovir; ABC, abacavir; NRTIs, nucleoside reverse transcriptase inhibitors; LPV, lopinavir; ATV, atazanavir; FosAPV, fosamprenavir; DRV, darunavir; SQV, saquinavir; NNRTIs, non-nucleoside reverse transcriptase inhibitors; RAL, raltegravir.

Variables with a normal distribution were described as mean (SD) and compared using the *t* test. Median and interquartile range (IQR) were employed to describe variables that did not follow a normal distribution, which were compared by using the Mann-Whitney non-parametric test. Percentages were compared using the χ^2^ square test or an exact binomial test when appropriate. The Kaplan-Meier method was used to calculate the time to virological failure and to treatment discontinuation. To evaluate factors associated with virological failure and development of dyslipidemia, we performed a Cox regression analysis and a multivariate logistic regression analysis, respectively. For the latter, we used only a subset of clinical and pharmacological variables to adjust the final multivariate model that avoided multi-collinearity. The hazard ratio and its 95% confidence interval (_95_CI) were also calculated. The statistical analysis was performed using SPSS version 15.0 (SPSS, Chicago, Illinois, USA). Differences were considered statistically significant at *p*<0.05.

**Figure 1:**
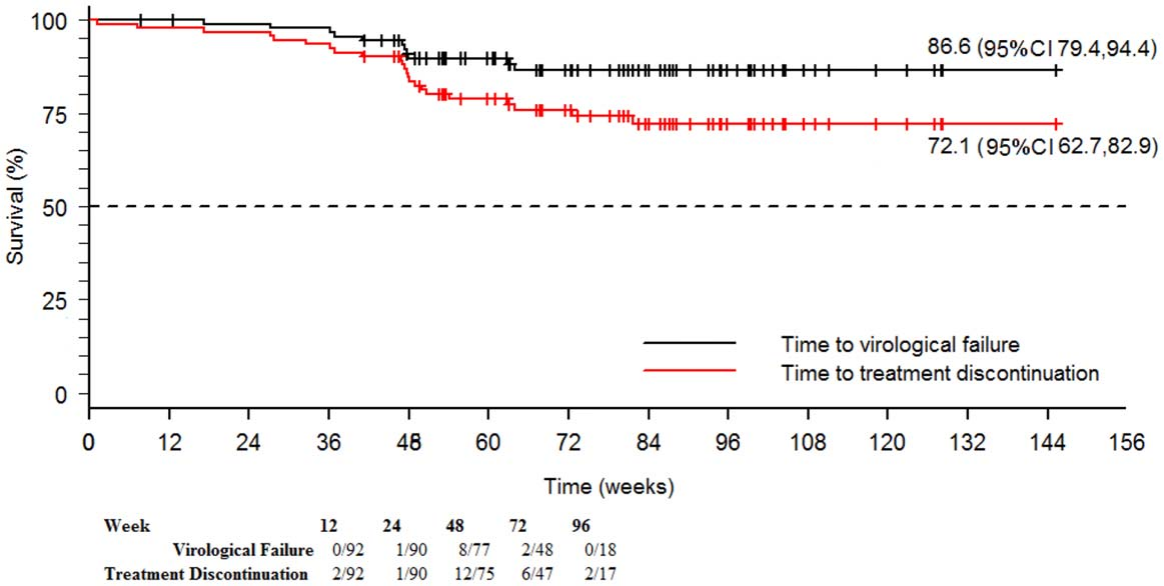
Time to virological failure and to treatment discontinuation in HIV-1-infected patients with DRV/r monotherapy. Abbreviations: 95% CI, 95% Confidence Interval. This figure shows the time to virological failure and time to treatment discontinuation for any reason among patients developing treatment failure during the overall follow-up.

## Results

### Baseline Characteristics

We identified 95 patients who were switched from their conventional ART regimen to DRV/r monotherapy while maintaining viral suppression. Three patients were excluded from the analysis because of voluntary interruption. Of the remaining 92 patients, the reasons for switching to DRV/r monotherapy were reduction in ART-related toxicity in 51 (55.4%) cases and patients’ request for simplification in 41 (44.6%) cases. The median (IQR) time to follow-up was 72.5 (57.1–92.3) weeks, and viral load had remained at <50 copies/mL for a median of 75.8 (32.4–175.8) weeks before DRV/r monotherapy was started. Patients had received a median of 5 (3–9) previous ART regimens. The regimen before monotherapy included lamivudine/emtricitabine (3TC/FTC) in 70 (76.1%) patients, TDF in 52 (56.5%), lopinavir/ritonavir (LPV/r) in 32 (34.8%), atazanavir/ritonavir (ATV/r) in 27 (29.3%), another boosted PI in 24 (26.1%), and non-NRTIs in 9 (9.7%) (Table 1).

**Figure 2:**
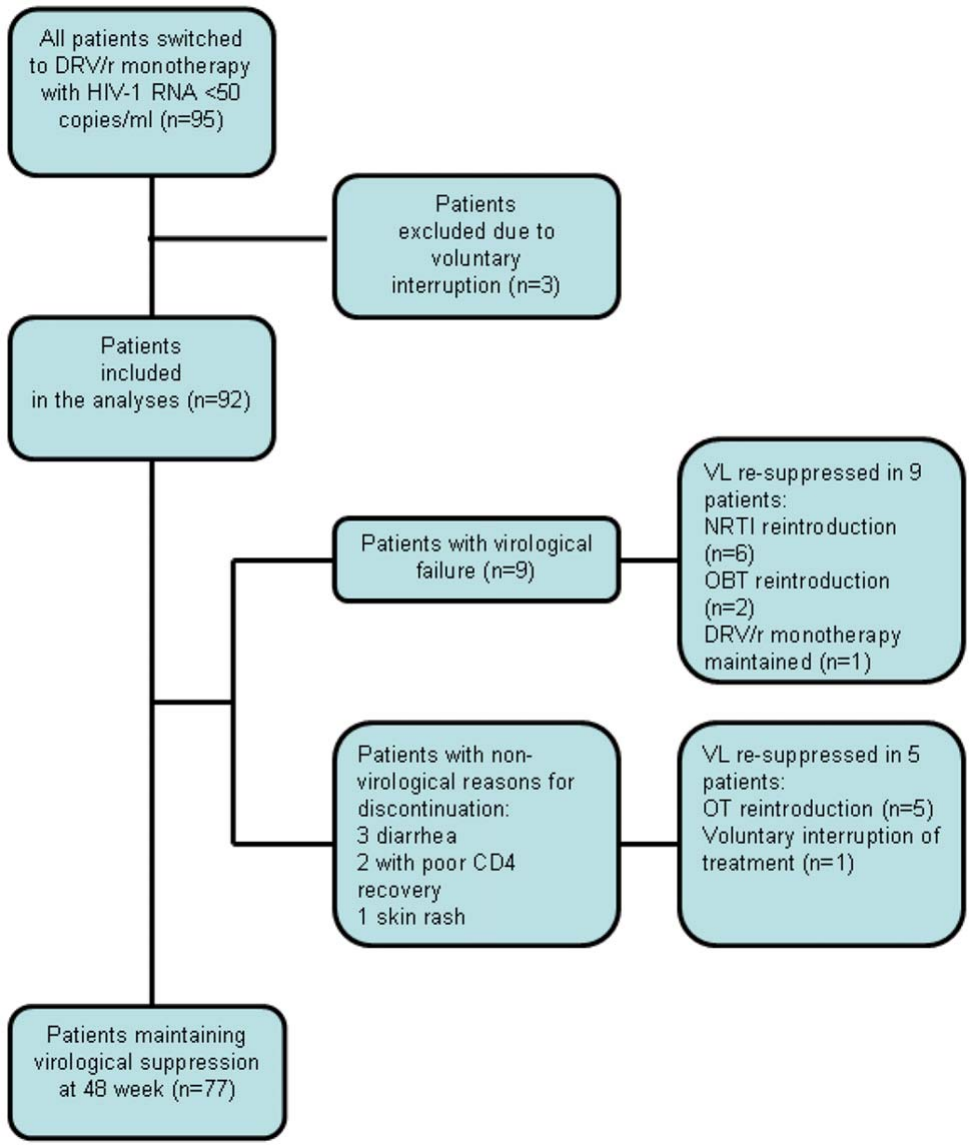
Summary of effectiveness results. Abbreviations: NRTI, nucleoside reverse transcriptase inhibitors; DRV/r, darunavir/ritonavir; OT, optimized treatment.

**Table 2:** Changes in laboratory parameters (n = 92).^a^

	Baseline	24 weeks	48 weeks	*P* value (Baseline vs48 weeks)
CD4+ T-cell count (cells/mm^3^)	604 (433–837)	629 (468–819)	595 (455.25–769.75)	0.327
Alkaline phosphatase (U/L)	79.5 (66–103)	64 (55–80.25)	64 (54.75–77.25)	<0.001
Aspartate aminotransferase (U/L)	23 (18–31)	22 (16–29)	22 (17–30)	0.352
Alanine aminotransferase (U/L)	22 (17–40.75)	23.5 (14–36.25)	23 (15–40.13)	0.317
Gamma-glutamyl transferase (U/L)	28 (19–38.75)	23 (17.25–36)	24 (18–35.13)	0.008
Creatinine (mg/100 mL)	0.89 (0.76–1.03)	0.89 (0.79–1.01)	0.85 (0.74–0.95)	<0.001

a All values are expressed as median (interquartile range).

**Figure 3:**
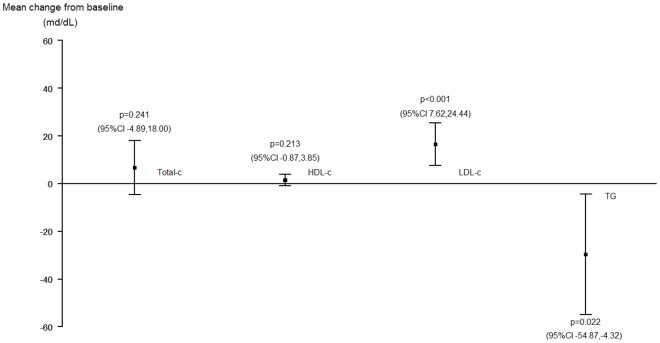
Changes in fasting lipid profile in patients switched to DRV/r monotherapy in routine clinical practice (n = 92). Abbreviations: Total-c, total-cholesterol; HDL-c, high-density lipoprotein cholesterol; LDL-c, low-density lipoprotein cholesterol; TG, triglycerides; 95% CI, 95% Confidence Interval.

**Table 3:** Factors associated with development of dyslipidemia.

	Risk analysis^a^							
	Total cholesterol ≥200 mg/dL		HDL-cholesterol <40 mg/dL		LDL-cholesterol ≥130 mg/dL		Triglycerides ≥200 mg/dL	
	OR (95% CI),univariate analysis	OR (95% CI), multivariateanalysis	OR (95% CI),univariate analysis	OR (95% CI), multivariate analysis	OR (95% CI), univariate analysis	OR (95% CI), multivariate analysis	OR (95% CI),univariateanalysis	OR (95% CI), multivariate analysis
Sex	1.682 (0.662,4.273)	–	0.593 (0.212,1.660)	–	1.261 (0.464,3.424)	–	2.385 (0.622,9.149)	–
Age (years)^b^	1.047 (0.998,1.097)	**1.041 (1.016,1.067)**	0.962 (0.913,1.014)	–	1.033 (0.983,1.087)	–	0.976 (0.924,1.031)	–
HCV	1.833 (0.678,4.957)	**0.256 (0.075,0.868)**	0.771 (0.261,2.284)	–	1.913 (0.665,5.503)	–	1.083 (0.311,3.773)	–
CD4+ T-cell count at baseline (every +100 cells/mm^3^)	1.000 (0.998,1.001)	–	1.001 (1.000,1.003)	–	1.000 (0.999,1.001)	–	1.000 (0.999,0.002)	–
Time since HIVdiagnosis (years)	1.028 (0.964,1.097)	–	1.006 (0.938,1.078)	–	1.028 (0.962,1.099)	–	0.977 (0.903,1.058)	–
Time on treatment (years)	1.046 (0.962,1.137)	–	0.946 (0.864,1.036)	–	1.013 (0.931,1.101)	–	0.984 (0.889,1.089)	–
No. of prior ARV regimens	1.075 (0.972,1.190)	–	0.996 (0.901,1.100)	–	1.065 (0.964,1.177)	–	1.029 (0.922,1.149)	–
ARV drugs used at entry								
TDF	1.278 (0.532,3.067)	–	1.259 (0.483,3.282)	–	1.169 (0.464,2.946)	–	1.607 (0.552,4.675)	–
ABC	0.267 (0.070,1.021)	–	4.237 (0.882,20.354)	–	0.517 (0.158,1.698)	–	1.994 (0.397,9.518)	–
LPV	1.294 (0.529,3.165)	–	0.863 (0.317,2.350)	–	0.888 (0.337,2.340)	–	1.066 (0.351,3.235)	–
ATV	1.249 (0.438,3.561)	–	0.143 (0.018,1.161)	–	1.438 (0.535,3.863)	–	2.232 (0.581,8.579)	–
DRV	2.611 (0.581,11.736)	–	0.321 (0.066,1.563)	–	0.871 (0.181,4.195)	–	0.370 (0.079,1.735)	–

Statistically significant results (p<0.05) are in bold.

Abbreviations: HDL, high-density lipoprotein; LDL, low-density lipoprotein; HCV, hepatitis C virus; ARV, antiretroviral; TDF, tenofovir; ABC, abacavir; NRTIs, nucleoside reverse transcriptase inhibitors; LPV, lopinavir;

ATV, atazanavir; DRV, darunavir; OR, odds ratio; CI, confidence interval.

a Univariate and multivariate logistic regression.

b Risk per year.

### Effectiveness

Nine (9.8%) patients experienced virological failure; 4 (44.4%) of these patients reported poor adherence. Among patients developing virological failure, the time to virological failure was 47.1 (IQR: 36.1–47.8; _95_CI 35.6, 58.5) weeks (Figure 1). Viral re-suppression was achieved in all cases either by reintroduction of the previous NRTI backbone (6 cases) or other antiretroviral drugs (2 cases). The remaining patient achieved viral load suppression <50 copies/mL on DRV/r monotherapy after adherence counseling.

Four (4.3%) patients discontinued DRV/r monotherapy because of adverse effects, and 2 (2.2%) experienced poor recovery of their CD4+ T-cell count. All these patients had viral load <50 copies/mL at the moment of DRV/r discontinuation. Virological suppression was maintained either by different antiretroviral regimens according to the clinician’s criteria (5 patients) or reintroduction of the NRTI backbone (1 patient). This last patient lost the virological suppression, because of voluntary treatment interruption. Finally, the time to treatment discontinuation for any reason was 48.5 (IQR: 28.9–53.2; 95CI 44.3, 51.6) weeks (Figure 1).

Thus, the overall proportion of patients who maintained viral suppression was 77/92 (83.7%), when only patients whose viral load was available up to week 48, and when patients who experienced DRV/r monotherapy discontinuation for any reason were considered. In the full dataset analysis, including the three patients with voluntary interruption, 77/95 (81.1%) of subjects maintained viral suppression. Figure 2 summarizes the effectiveness results.

Seventeen (18.5%) patients developed blips (HIV RNA <200 copies/mL) at a median (IQR) of 45 (29.8–49.5) weeks after DRV/r monotherapy switching. None of these patients experienced virological failure.

Virological failure was not significantly associated to any of the factors analysed in the univariate or multivariate analysis (i.e., presence of blips, gender, age, HCV co-infection, CD4+ nadir, CD4+ T-cell count at baseline, CDC stage, time since HIV diagnosis, time on ART, duration of virological suppression, and number of prior PIs and antiretroviral regimens).

### Genotyping

Genotyping data prior to DRV/r monotherapy were available for 31 (29%) patients. All these patients had viral strains with polymorphic or minor PI resistance–associated mutations (median number of mutations, 4 [IQR: 2–5]). Three patients had major mutations in the protease gene (one patient had L90M, another D30N, and the third M46I and V82A), which were not associated with resistance to DRV. None of the patients experienced virological failure during follow-up.

Genotyping data were available at virological failure in 3/9 patients. One patient had no protease gene mutations, whereas the other 2 showed the following mutations: R41K, I62V, L63P, I72V/I, V77I, and I93L; and L33V, E35D, D60E, and I64V, respectively. Although no previous genotypes were available to establish comparisons, none of these mutations have been described to be associated with DRV resistance.

### Safety

The changes observed in the median values for CD4+ T-cell count, aspartate aminotransferase, and alanine aminotransferase at week 48 of follow-up were not significant (*p* = 0.327, *p* = 0.352, and *p* = 0.317, respectively). However, a significant reduction was observed in the values for alkaline phosphatase, gamma-glutamyl transferase, and creatinine (*p*<0.001, *p* = 0.008, and *p*<0.001, respectively) (Table 2).

Four (4.3%) patients discontinued DRV/r monotherapy because of adverse events and 2 (2.2%) experienced poor recovery of their CD4+ T-cell count. Adverse events leading to discontinuation of DRV/r included diarrhea in 3 patients and skin rash in 1 patient that resolved with voluntary interruption of DRV/r monotherapy. It is noteworthy that 2 of these 4 patients had not received PI-based regimens at baseline.

### Fasting Lipid Profile

Overall, the median (IQR) LDL-cholesterol level increased significantly from 116.05 (82.5–137.5) mg/dL at baseline to 137.3 (101.1–155.2) mg/dL at week 48 of follow-up (*p* = 0.001), with a median increase of 13.0 (−5.0 to 35.0) mg/dL. In addition, triglyceride levels decreased significantly by a median of −17.6 (−53.4 to 26.2) mg/dL. There were no significant changes in total cholesterol (*p* = 0.241) or HDL-cholesterol (*p* = 0.213) (Figure 3).

A more sensitive analysis based on previously used antiretroviral drugs showed significant increases in total-cholesterol from 176.1 (150.9–212.8) to 210.6 (178.9–238.9) mg/dL (*p* = 0.001), and LDL-cholesterol levels from 103.7 (80.2–127.0) to 115.0 (101.1–155.5) mg/dL (*p*<0.001) in patients with prior use of TDF. On the contrary, in patients who had recently received ABC, total cholesterol at baseline was 205.1 (191.5–230.2) mg/dL and 211.1 (200.1–233.1) mg/dL at week 48 (*p* = 0.985), and LDL-cholesterol changed from 127.3 (109.2–153.2) to 139.0 (125.7–153.1) mg/dL (*p* = 0.566). A significant decrease in total cholesterol from 220.6 (192.1–277.6) to 201.2 (182.0–232.2) mg/dL and triglyceride levels from 208.5 (133.0–348.4) to 142 (100.0–177.0) mg/dL was observed in patients who had recently received LPV/r (*p* = 0.028 and *p*<0.001, respectively). In patients who had recently received ATV/r, a significant increase was observed in total cholesterol from 185.8 (151.0–216.7) to 213.0 (173.9–249.6) mg/dL (*p* = 0.016); in patients who had recently received saquinavir, a significant increase was observed in total cholesterol from 197.3 (170.2–218.6) to 243.8 (203.1–267.0) mg/dL and LDL-cholesterol levels from 120.3 (90.9–131.5) to 151.0 (116.5–154.7) mg/dL (*p* = 0.049 and *p* = 0.011, respectively).

According to the ATP III classification [11], there were no significant changes in the percentage of patients with different cut-off levels for cholesterol (total, LDL, and HDL) and triglycerides (*p*>0.05 in all analysis). In addition, the percentage of patients taking lipid-lowering drugs increased from 12% to 26% (*p* = 0.042).

In the univariate logistic regression analysis, no factors were associated with the development of dyslipidemia. Nevertheless, in order to find a subset of variables that would account for the development of dyslipidemia, a multivariate analysis (OR [95% CI]) including all variables was performed. Only age was associated with increased total cholesterol (OR = 1.041 [1.016,1.067]), and HCV co-infection was found to be associated with a lower risk of hypercholesterolemia (OR = 0.256 [0.075,0.868]). There was no association between antiretrovirals used before the switch to DRV/r and development of dyslipidemia (Table 3).

## Discussion

This study shows that simplification of ART with DRV/r monotherapy in routine clinical practice is effective and well tolerated in HIV-1-infected individuals with sustained viral suppression. Although DRV/r monotherapy was also associated with a significant increase in overall LDL-cholesterol levels, a significant decrease in total cholesterol and triglyceride levels was observed in patients with prior LPV/r. Therefore, with the possible exception of patients with LPV/r-associated dyslipidemia, DRV/r monotherapy does not seem to confer a clear advantage in the routine management of dyslipidemia.

In the MONET study, DRV/r monotherapy was non-inferior to DRV/r plus 2 NRTIs at 48 weeks in all analyses (per protocol, intent-to-treat switch equals failure, and switch-included analysis) [7]. However, at 96 weeks, DRV/r monotherapy retained non-inferiority only in the switch-included and observed-failure analyses, but not in the main switch-equals-failure analysis [12]. In the MONOI study, non-inferiority to DRV/r plus 2 NRTIs was only observed in the per-protocol analysis, but not in the intention-to-treat analysis. The results of both analyses were concordant for the magnitude of difference in efficacy between arms, but discordant for the non-inferiority margin [8].

In our cohort, some baseline characteristics are different in comparison to MONET and MONOI trials. Our cohort included patients with slightly longer times since HIV diagnosis and previous exposure to ART, a higher proportion of HCV-coinfected patients, more heterogeneous treatment exposure with more PI experience, and less NNRTI use at baseline than those included in previous clinical trials [7], [8]. Despite these differences, the effectiveness rates of DRV/r monotherapy were similar to those found in the MONET and MONOI studies [7], [8]. At least half of those patients with detectable viremia in our analysis reported suboptimal adherence. In addition, as observed in clinical trials [7], [8], the development of virological failure to DRV/r monotherapy in our cohort was not associated with the loss of future therapeutic options, it was rarely associated with resistance to PIs, and reintroduction of NRTIs achieved virological suppression in all patients with virological failure.

Based on preliminary data suggesting a potential benefit of PI monotherapy in the setting of NRTI-related toxicity [13], [14], the latest antiretroviral treatment guidelines from the International Antiviral Society-USA (IAS-USA) and the European AIDS Clinical Society (EACS) recognize PI monotherapy as an alternative NRTI-sparing strategy if NRTI-related toxicity or intolerance develops [3], [4].

We found that DRV/r monotherapy was well tolerated: only 4 patients (4.3%) discontinued therapy owing to adverse events, which were mostly gastrointestinal. Moreover, the significant reductions in creatinine and alkaline phosphatase values observed in these patients suggest that DRV/r monotherapy could potentially improve TDF-associated kidney and bone toxicity.

Of note, lipid disorders associated to the use of ART could be considered as toxic effect that leads to the use of PI monotherapy. However, data on the effect of PI monotherapy on lipid profile are scant. The most common laboratory abnormality observed in patients using DRV/r monotherapy in the MONET study was an increase in total cholesterol, particularly in patients who interrupted TDF at baseline. Additionally, individuals starting TDF in the triple therapy arm showed decreases in total cholesterol [7]. Alongside other evidence [15]–[17], this has led some authors to suggest a lipid-lowering effect of TDF. However, is noteworthy that the increase in total cholesterol observed in the MONET study was only observed in 8 patients (5 in DR/r monotherapy group and 3 in triple therapy arm) [7]. So, conclusions about the role of DRV/r monotherapy in the setting of lipid disorders could not be properly provided.

We found initiation of DRV/r monotherapy to be associated with a significant increase in LDL and total cholesterol levels, as well as with an increase in the number of patients taking lipid-lowering therapy. In agreement with MONET study, this was also particularly evident in patients who withdrew TDF at baseline [7]. As previously reported [18]–[20], younger age and HCV co-infection were associated with a decreased risk of developing hypercholesterolemia in the multivariate analysis. On the other hand, triglyceride and total cholesterol levels improved in patients who switched from triple therapy regimens including LPV/r to DRV/r monotherapy. These changes, however, were not reflected in significant changes in the proportion of patients with dyslipidemia according to the ATP III classification. Perhaps with the exception of patients previously receiving LPV/r, our results suggest that DRV/r monotherapy does not confer a clear advantage in the management of metabolic disorders.

The main limitations are the relatively low number of patients included, the retrospective design, which could lead to bias or unmeasured confounding, and the lack of a comparative arm to assess the magnitude of differences relative to continuing standard ART. Currently, PI monotherapy, particularly with DRV/r, has a defined use in highly selected patients, so large cohort studies are difficult to perform. To our knowledge, there is only one large ongoing long-term cohort study of patients with PI monotherapy (Protease Inhibitor Monotherapy Versus Ongoing Triple-therapy in the Long Term Management of HIV Infection (PIVOT). Avalible at: http://clinicaltrials.gov, identifier NCT01230580), but the communication of the results is not foreseen in short term. Therefore, our study is the first to report the safety and virological effectiveness of DRV/r monotherapy in routine clinical practice and our findings provide insight into the advantages and disadvantages, especially those related to metabolic effects of using DRV/r monotherapy in daily clinical practice and, thus, useful information for future clinical trials.

In conclusion, DRV/r monotherapy appears to be safe and effective in HIV-1-infected patients with sustained viral suppression in routine clinical practice. However, with the possible exception of patients previously receiving LPV/r, DRV/r monotherapy does not seem to confer a clear benefit in the management of dyslipidemia. Prospective controlled studies are needed to elucidate the effects of DRV/r monotherapy on cholesterol levels, as well as on the management of antiretroviral-related kidney and bone toxicity.
